# Race Analysis of the World’s Best Female and Male Marathon Runners

**DOI:** 10.3390/ijerph17041177

**Published:** 2020-02-13

**Authors:** Véronique Billat, Damien Vitiello, Florent Palacin, Matthieu Correa, Jean Renaud Pycke

**Affiliations:** 1 Université de Paris, EA3625-Institut des Sciences du Sport Santé de Paris (I3SP), 75015 Paris, France; damien.vitiello@parisdescartes.fr (D.V.); palacinflorent@gmail.com (F.P.); matthieucorrea37@gmail.com (M.C.); jeanrenaud.pycke@univ-evry.fr (J.R.P.); 2 Véronique Billat, Université de Paris, EA 3625-Institut des Sciences du Sport Santé de Paris (I3SP), 1 rue Lacretelle, 75015 Paris, France; 3 Université d’Evry Val d’Essonne, UMR8071—CNRS-Laboratoire de Mathématiques et Modélisation d’Evry (LaMME), 91037 Evry, France

**Keywords:** running strategy, critical speed, endurance, performance, health

## Abstract

**Background:** Beyond the difference in marathon performance when comparing female and male runners, we tested the hypothesis that running strategy does not different according to sex. The goal of the present study is to compare the running strategy between the best female and male marathon performances achieved in the last two years. **Methods:** Two aspects of the races were analyzed: (i) average speed relative to runner critical speed (CS) with its coefficient of variation and (ii) asymmetry and global tendency of race speed (i.e., the race’s Kendall τ). **Results:** The females’ best marathons were run at 97.6% ± 3% of CS for the new record (Brigid Kosgei, 2019) and at 96.1% ± 4.4% for the previous record (Paula Radcliffe, 2003). The best male performances (Eliud Kipchoge, 2018 and 2019) were achieved at a lower fraction of CS (94.7% ± 1.7% and 94.1% ± 2.3% in 2018 and 2019, respectively). Eliud Kipchoge (EK) achieved a significant negative split race considering the positive Kendall’s τ of pacing (i.e., time over 1 km) (τ = 0.30; *p* = 0.007). Furthermore, EK ran more of the average distance below average speed (54% and 55% in 2018 and 2019, respectively), while female runners ran only at 46% below their average speed. **Conclusions:** The best female and male marathon performances were run differently considering speed time course (i.e., tendency and asymmetry), and fractional use of CS. In addition, this study shows a robust running strategy (or signature) used by EK in two different marathons. Improvement in marathon performance might depend on negative split and asymmetry for female runners, and on higher fractional utilization of CS for male runners.

## 1. Introduction

For 20 years, marathon racing has gained popularity given that it is one of the rare sporting events in which elite and non-elite runners compete at the same time, despite some athletes completing the race in twice the time of others. Although males are nearly able to finish the race in under two hours (Eliud Kipchoge (EK): 2 h 01 min 39 s in Berlin, 2018, and 2 h 02 min 37 s in London, 2019), the milestone for female runners of 2 h 15 min was broken by Brigid Kosgei (BK) (2 h 14 min 04 s) in 2019 during the Chicago marathon, 16 years after the previous world record of Paula Radcliffe (PR) at the London marathon in 2003.

Beyond speculation on the future of performance or on the comparison of relative performance in males and females [1,2], the goal of this study is to test the hypothesis that running strategy does not differ according to sex. We consider that both female and male marathoners might benefit from a training program based on their perception to improve their performance. Indeed, this may allow them to have a more positive asymmetry, a lower coefficient of variation of speed, and to run at a higher percentage of their critical speed (CS) during the entire race. Therefore, this study analyzed two aspects of the races: (i) the average runner’s speed relative to their CS [3,4,5], with the coefficient of variation and (ii) the tendency of the pace and its asymmetry [6].

## 2. Materials and Methods

To achieve this study, pacing (i.e., time per distance) run by EK (35 years old, Berlin 2018 and London 2019), BK (25 years old, Chicago 2019) and PR (29 years old, London 2003) were examined. Data were retrieved from the World Athletics website on 14th October 2019. A computation of the average speed per distance was achieved by dividing distance per time unit.

### 2.1. Critical Speeds (CS)

The average speed relative to runner CS, with the coefficient of variation, was calculated. CS was calculated from the runner’s personal best performances in the 3000 m and half marathon (run in less than 1h for EK, and 1 h 04 min 28 s and 1 h 05 min 40 s for BK and PR, respectively).

The CS was calculated using the following equation [7]:

(1) $$D_{lim}=\;\alpha +\;\beta \;t_{lim}$$

$D_{lim}$ = distance; α = constant reserve; $\beta =\mathrm{critical}\;\mathrm{speed};\;t_{lim}$ = record time.

### 2.2. Global Tendency of Pace and Its Asymmetry

Here, the trend in speed time series (i.e., Kendall’s τ non-parametric rank correlation coefficient) [8] and the pacing design (i.e., asymmetry characteristics of the race) [6] were compared. Here is the equation of Kendall’s τ:

(2) $$\tau =2\;/\;n\left(n\;-\;1\right)\;\overset{}{\underset{i<j}{\sum }}K\left(vi,\;vj\right)$$

vi = *i*th value of a speed; vj = *j*th value of a speed; *i* < *j* = *i* indicates a period of time prior to *j*; sum being performed over the n(n − 1)/2 distinct unordered couples of indices {*i*, *j*}, so that τ takes values in between −1 and 1.

We sought to establish running strategy or signature in real race format for the same runner (EK), and for male and female official best marathon performances.

## 3. Results

### 3.1. Average Marathon Speed Relative to Marathoners’ Critical Speed and Coefficient of Speed Variation

The male and female marathon races were run at different percentages of the CS (Table 1).

Indeed, the best female marathon performances were run at 97.6% ± 3% of CS for the new record (BK, 2019) and 96.1% ± 4.4% for the previous record (PR, 2003), while the best male performance (i.e., EK, Berlin 2018) was run at a lower fraction of CS (94.7% ± 1.7%).

### 3.2. Speed Trend and Asymmetry Characteristics

**Figure 1:**
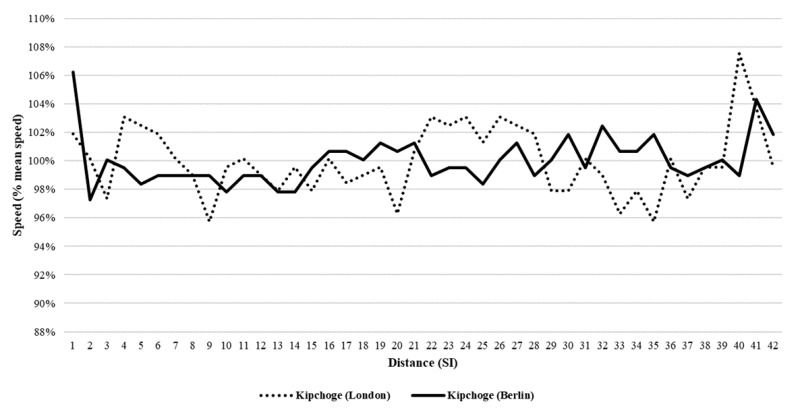
Speed trend and asymmetry characteristics in the world’s fastest male marathoner. The curve (dotted line) represents the time course of Eliud Kipchoge’s running speed during the London marathon in 2019. The curve (dashed line) represents the time course of his running speed during the Berlin marathon in 2018. These curves represent the percentage of the mean speed achieved in each distance unit during the entire marathon.

**Figure 2:**
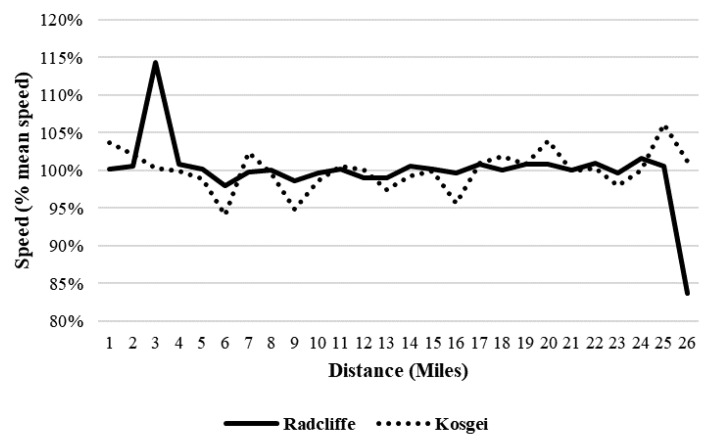
Speed trend and asymmetry characteristics in the world’s fastest female marathoners. The curve (solid line) represents the time course of running speed during the previous world record marathon reached by Paula Radcliffe during the London marathon in 2003. The curve (dotted line) represents the time course of running speed during the new world record reached by Brigid Kosgei during the Chicago marathon in 2019. These curves represent the percentage of the mean speed achieved in each distance unit during the entire marathon.

EK (male athlete) achieved a large negative split race considering pace with a positive Kendall’s τ (0.30; *p* = 0.007) and ran more of the average distance below average speed (Figure 1) (54%). By contrast, there were no trends in previous and current marathon world records of BK and PR (female athletes) (Figure 2). Their races were not optimal in regard to speed asymmetry as BK (66%) and PR ran 46% of the distance above their average speed.

## 4. Discussion

To the best of our knowledge, this is the first study to compare the running strategy between the best male and female marathoners, based on a previous statistical analysis jointly using the trend and asymmetry of the race [6]. In this study, it was demonstrated that EK, PR, and BK (i) did not use the same running strategy and (ii) did not use the same fraction of their respective CS. Finally, EK achieved his two best performances using the same running signature on two different marathon routes (2 h 01 min 39 s in Berlin 2018, and 2 h 02 min 37 s in London 2019).

This study demonstrated that females ran at a constant pace considering their Kendall’s τ was between −0.05 and 0.05 [6]. It was also highlighted that world records are broken using a running strategy based on running speeds below median speed, unlike popular marathoners who run more distance at speeds above the median [9]. This may be due to the fact that lower level runners (>2 h 20 min) run at too high a target that they cannot maintain beyond the 26th km, where the average speed (i.e., final performance) is reduced and is therefore lower than the median speed. These results may ask questions of the perception of physiological load, the plan of action to produce speed variations (or not), and the strategy of the U-race, which is conducive to performance but which requires a high reserve of power. This latter point may imply training protocols based on running acceleration and deceleration [10]. Therefore, high intensity interval training using both positive and negative acceleration to attain VO_2_max at a wide range of speeds may allow an increase of endurance capacity and anaerobic power, which are required to achieve top performances in the marathon [11,12,13].

The present work also examined the newly established marathon world records (in 2019, for both EK and BK) using an assessment of the race speed asymmetry [6]. We also examined pacing variability and confirmed that this was run at and below 3% in marathons [14]. The speed coefficient of variation of EK was within 1.7%, indicating a relative pacing strategy. In addition, the asymmetry of the speed time series of the runners was also analyzed. Our results suggest the possibility of establishing a world record (i.e., Berlin 2018) by running below the average pace, thanks to a very fast start and finish, in the classical format of the marathon.

Finally, the marathon should not be a constant speed race [15] and new strategies of running must be addressed to allow better performance in the future. As VO_2_max is not sufficient to distinguish high level marathon runners (2 h 11 min–2 h16 min) from elite marathon runners (less than 2 h 11 min) [16], this parameter may be used in parallel with average running speed and CS to propose a new pacing strategy adapted to the level of marathoners. Indeed, this study highlighted that average speed represents 94% of CS. The value is close to personal best one-hour record, meaning that the runner might be able to double his time limit just by decreasing his speed by 5%. Finally, another way of optimizing the running strategy might be the development of runners’ anaerobic capacity and their ability to use a higher fraction of their CS during the marathon.

## 5. Practical Applications

- A new training program based on athletes’ perception could be introduced to better adapt their speed while running a marathon.
- New training methods in marathon running could optimize the running strategy to allow the marathoner to have a positive asymmetry and a lower coefficient of variation of speed during the race.
- The CS is a crucial parameter that might be improved by new training methods since a high fraction of the CS is achieved in both female and male marathon world records.
- A performance target for coaches and athletes should be the maintenance of constant pace just below average pace and at 95% of the CS.

## 6. Conclusions

Due to the already high fraction of CS achieved in marathons by females and also for the world’s best performance by EK, this parameter and the average speed achieved in each km might be two parameters to consider in elaborating a new running strategy to improve performance in marathon and short events, as already reported [16]. In addition, optimizing the running strategy with positive asymmetry and lower coefficient of variation of speed might allow marathoners to run more slowly at almost a constant pace, just below the average pace, and at 95% of CS in accordance with their higher speed reserve. However, future research on other marathoners is necessary to confirm difference in running strategy between females and males and to verify consistency of running signature for a single marathon.

## Figures and Tables

**Table 1:** Average marathon speed relative to male and female marathoners’ critical speed and coefficient of speed variation.

			Speed			Critical Speed (km)	Speed (% Critical Speed)			Skewness (% km below Mean Speed)	Pace/Trend	
Athlete	Place/Year	Time	Mean	SD	Variation Coefficient		Mean	SD	Variation Coefficient		Kendall’s t	*p*-Value
E. Kipchoge	London/2019	2 h 02 min 37 s	20.67	0.51	2.48%	21.6	94.1	2.3	2.44%	55	−0.0289	0.8000
E. Kipchoge	Berlin/2018	2 h 01 min 39 s	20.78	0.35	1.68%	21.6	94.7	1.7	1.80%	54	0.3000	0.0069
P. Radcliffe	London/2003	2 h 15 min 25 s	18.89	0.30	1.59%	19.6	96.0	4.4	4.58%	32	0.0050	0.7227
B. Kosgei	Chicago/2019	2 h 14 min 04 s	18.82	0.65	3.45%	19.29	97.6	3.4	3.45%	46	0.0955	0.4891

Data are presented in means ± SD and percentages.
